# Strong Family-Level Clustering of Adolescent Weight Status Despite Limited Associations with Individual Parental Anthropometric and Metabolic Characteristics

**DOI:** 10.3390/jcm15155790

**Published:** 2026-07-24

**Authors:** Vasilios Mournos, Georgios Dimakopoulos, Theano Makridou, Konstantinos Kontotasios, Dimitri J. Pournaras, Carel W. le Roux, Alexander Kokkinos, Kalliopi Kotsa, Theocharis Koufakis

**Affiliations:** 1 Medical School, Aristotle University of Thessaloniki, 54124 Thessaloniki, Greece; basilismournos1998@gmail.com; 2 BIOSTATS, Epirus Science and Technology Park Campus, University of Ioannina, 45110 Ioannina, Greece; info@biostats.gr; 3 General Hospital of Veria, 59132 Veria, Greece; 4 North Bristol NHS Trust, Bristol BS10 5NB, UK; 5 Population Health Sciences, Bristol Medical School, University of Bristol, Bristol BS8 1UD, UK; 6 School of Medicine, University College Dublin, DO4 V1W8 Dublin, Ireland; 7 First Department of Propaedeutic Internal Medicine, Medical School, National and Kapodistrian University of Athens, Laiko General Hospital, 11527 Athens, Greece; rjd@otenet.gr; 8 Division of Endocrinology and Metabolism and Diabetes Center, First Department of Internal Medicine, Medical School, Aristotle University of Thessaloniki, AHEPA University Hospital, 54636 Thessaloniki, Greece; 9 Second Propaedeutic Department of Internal Medicine, Medical School, Aristotle University of Thessaloniki, Hippokration General Hospital, 49 Konstantinoupoleos Str., 54642 Thessaloniki, Greece

**Keywords:** adolescent obesity, body mass index, family clustering, anthropometric characteristics

## Abstract

**Objectives**: Familial aggregation of obesity has been consistently reported; however, previous studies have predominantly relied on body mass index (BMI)-based associations and have dedicated insufficient time to examining the contribution of measurable parental biological traits to family-level clustering. We investigated associations between parental anthropometric and metabolic characteristics and adolescent weight status in families in which at least one parent had obesity, while exploring family-level clustering patterns. **Methods**: This cross-sectional family-based study included 34 families comprising 110 individuals (65 parents and 45 adolescents) consecutively recruited from primary care registries. Participants underwent anthropometric and biochemical evaluation, including indices of central body fat distribution. Linear mixed models were used to investigate associations between parental characteristics and adolescent BMI percentile while accounting for non-independence of observations within families. **Results**: Mean parental BMI was 31.37 ± 5.67 kg/m^2^, while 61.5% of parents had obesity. Among adolescents, 28.9% had BMI percentile between the 85th and 95th percentiles and 13.3% had a BMI percentile > 95th percentile. Strong family-level clustering of adolescent weight status was consistently observed across analyses, with intraclass correlation coefficients ranging from 0.716 to 0.728. In contrast, parental characteristics demonstrated limited explanatory contribution (marginal R^2^ 0.028–0.044), while no isolated parental variable showed a significant association with adolescent BMI percentile. **Conclusions**: Strong family-level clustering of adolescent weight status was observed despite the absence of significant associations with individual parental anthropometric and metabolic characteristics, suggesting that familial obesity susceptibility may reflect shared influences extending beyond routinely measurable traits alone.

## 1. Introduction

Obesity is increasingly recognized as a complex, chronic, relapsing disease that results from the interaction of genetic, biological, behavioral, environmental, and societal determinants rather than a simple imbalance between energy intake and expenditure. Its rapidly increasing prevalence has made obesity one of the greatest global public health concerns, with profound implications for healthcare systems and population health [1]. Although effective therapeutic options have expanded considerably in recent years, prevention remains a fundamental priority. A better understanding of the determinants of obesity susceptibility, particularly during critical stages of development, is therefore essential to inform targeted prevention strategies and identify individuals at increased risk before obesity becomes established.

Childhood and adolescent obesity represent major global public health challenges with continuously increasing prevalence over recent decades [2]. Excess adiposity during early life is associated with increased risk of cardiometabolic disease, type 2 diabetes, hypertension, dyslipidemia, metabolic dysfunction-associated steatotic liver disease, and premature cardiovascular morbidity in adulthood [3]. Importantly, obesity during adolescence frequently persists into adult life, emphasizing the need for early identification of high-risk populations and a better understanding of the factors contributing to obesity susceptibility within families [4].

Familial aggregation of obesity has been consistently reported in epidemiological studies, suggesting that parental adiposity substantially influences body weight trajectories in offspring [5]. Both environmental and biological mechanisms have been proposed to contribute to this association [6,7]. Large population-based family studies have consistently demonstrated that parental body mass index (BMI) is among the strongest predictors of childhood and adolescent adiposity [8]. Nevertheless, considerable heterogeneity in offspring weight status persists even among families with parental obesity, suggesting that BMI alone may not fully capture the biological complexity underlying familial obesity susceptibility.

Most previous studies investigating the relationship between parental and childhood obesity have focused predominantly on BMI-based classifications and pairwise associations between parental and offspring anthropometric measures [9]. Although BMI remains the most widely used anthropometric measure and one of the strongest predictors of offspring adiposity, it does not distinguish between fat and lean mass or adequately reflect body fat distribution or metabolic health [10]. In contrast, indices of central adiposity, including waist circumference, waist-to-height ratio (WHtR) and waist-to-hip ratio (WHR), provide a closer approximation of visceral fat accumulation, which is more strongly associated with insulin resistance and cardiometabolic risk than BMI alone [11]. Similarly, routinely measured metabolic biomarkers, including fasting glucose, triglycerides and high-density lipoprotein cholesterol, may identify early metabolic dysfunction and adverse cardiometabolic phenotypes among individuals with comparable BMI [12]. We therefore hypothesized that these routinely available parental characteristics might provide complementary information beyond BMI in characterizing familial obesity susceptibility. However, their contribution has not been adequately investigated within family-based hierarchical analytical models. Furthermore, evidence from Mediterranean and specifically Greek populations remains relatively scarce despite the high prevalence of adolescent and adult obesity in the region [13].

The aim of the present study was to investigate the relationship between parental anthropometric, metabolic, and clinical characteristics and obesity-related parameters in adolescents from families in which at least one parent fulfilled BMI criteria for obesity. In addition, we sought to explore the presence of family-level clustering patterns of adolescent BMI using a hierarchical analytical approach accounting for within-family structure.

## 2. Methods

### 2.1. Study Design and Population

This cross-sectional family-based study included families recruited consecutively from a large general practitioner (GP) registry network in Northern Greece incorporating five smaller primary care units. The study population was relatively ethnically homogeneous and consisted predominantly of families of Greek origin from mixed urban and rural communities of low-to-middle socioeconomic background. Families were enrolled consecutively between December 2025 and February 2026 upon fulfilling eligibility criteria during routine clinical follow-up in the primary care setting. The study population consisted of biological parents and adolescents aged 12–18 years. Families were eligible if at least one parent fulfilled the World Health Organization BMI criteria for obesity. This eligibility criterion was chosen to enrich the study population for familial obesity risk and to explore determinants of variability in adolescent weight status within a high-risk familial setting. In families with two available parents, data from both parents were collected. Adolescents were included irrespective of BMI status. In adults, BMI 18.5–24.9 kg/m^2^ was considered normal body weight, BMI 25.0–29.9 kg/m^2^ overweight, and BMI ≥ 30 kg/m^2^ obesity [14]. In adolescents, weight classification was based on age- and sex-specific BMI percentiles, with values < 85th percentile considered normal body weight, between the 85th and 95th percentiles overweight, and >95th percentile obesity [15].

Exclusion criteria included inability to provide informed consent, absence of available anthropometric measurements, acute illness at the time of assessment, and known medical conditions expected to substantially affect body composition or linear growth independently of obesity. Clinically stable treated hypothyroidism was not considered an exclusion criterion. All participating families had previously received standard lifestyle counseling as part of routine primary care management by the same GP.

### 2.2. Anthropometric, Clinical and Laboratory Assessment

Anthropometric assessment was performed using standardized procedures during routine clinical visits. Body weight was measured with participants wearing light clothing and no shoes. Height was measured using a wall-mounted stadiometer. BMI was calculated as body weight in kilograms divided by height in meters squared (kg/m^2^) [16]. Waist circumference was measured at the midpoint between the lower rib margin and the iliac crest, while hip circumference was measured at the level of the greater trochanters [17]. Neck circumference was measured at the midpoint of the neck. WHR and WHtR were subsequently calculated [18]. WHR and WHtR were selected because they are simple, widely validated surrogate markers of central adiposity that are routinely used in clinical practice and have established associations with cardiometabolic risk. More complex composite adiposity indices were not evaluated because the study aimed to focus on routinely available clinical measurements applicable to real-world primary care settings. The body fat percentage of adult participants was estimated using the United States Navy circumference-based anthropometric formula [19]. Blood pressure measurements were obtained after a resting period using appropriately sized cuffs according to standard clinical practice [20]. All measurements were independently confirmed by a second investigator. Medical history and obesity-related diseases were recorded based on participant self-report and review of available medical records.

Venous blood samples were collected after an overnight fast and all laboratory measurements were performed in the same laboratory. Laboratory assessment included hematological parameters, liver enzymes, renal function indices, fasting glucose, glycated hemoglobin (HbA1c), and lipid profile measurements. The estimated glomerular filtration rate (eGFR) was calculated using the Chronic Kidney Disease Epidemiology Collaboration equation [21], while the fibrosis-4 (FIB-4) score was calculated in parents using the standard formula [22].

### 2.3. Statistical Analysis

Continuous variables are presented as mean ± standard deviation, whereas categorical variables are presented as absolute frequencies and percentages. To investigate the association between parental characteristics and BMI percentile in adolescents, linear mixed models (LMMs) were constructed using adolescent BMI percentile as the dependent variable. Separate univariate models were fitted for each parental anthropometric, biochemical, hematological, and clinical parameter. A multivariable mixed-effects model was not constructed because the primary objective was to evaluate the explanatory contribution of individual routinely measured parental characteristics relative to family-level clustering. In addition, the limited sample size and the substantial collinearity among several anthropometric and metabolic variables were considered likely to produce unstable parameter estimates and overfitting in a multivariable model.

Parental age and sex were included as fixed covariates in all models. Family identification number was incorporated as a random intercept to account for within-family clustering and non-independence of observations arising from the inclusion of parents and siblings within the same family structure. An initial model incorporating both family and child identification numbers as random intercepts was attempted; however, the model failed to converge, likely due to insufficient cluster size, and was therefore simplified to a single family-level random intercept. Degrees of freedom were estimated using the Satterthwaite approximation. Regression coefficients (B), standard errors (SE), intraclass correlation coefficients (ICC), marginal R^2^, and conditional R^2^ values were calculated for each model. Ninety-five percent confidence intervals (CIs) for ICC estimates were obtained using parametric bootstrap with 1000 iterations, as implemented in the lme4 package in the R statistical environment. The significance of random effects was assessed using likelihood ratio testing. A two-sided *p* value < 0.05 was considered statistically significant.

Statistical analyses were performed using the Jamovi statistical environment (version 2.5; The jamovi project, 2024), with LMMs fitted via the GAMLj module implementing the lme4 package in the R statistical environment (R Foundation for Statistical Computing, Vienna, Austria).

### 2.4. Ethics

The study protocol was approved by the Bioethics Committee of Aristotle University of Thessaloniki (approval code: 45/approval date: 2 December 2025) prior to study initiation. Written informed consent was obtained from all adult participants. In the case of minors, written informed consent was obtained from parents or legal guardians, while assent was also obtained from adolescent participants. The study was conducted in accordance with the principles of the Declaration of Helsinki. Artificial intelligence (AI)-based tools (ChatGPT, v. 5.5, OpenAI, San Francisco, CA, USA) were used to assist exclusively with language polishing and figure creation. No AI system was involved in study design, data collection, analysis, or interpretation. All scientific content, conclusions, and responsibility for the work remain entirely with the authors.

## 3. Results

### 3.1. Study Population

The study included 34 families comprising 65 parents and 45 adolescents. Among parents, 31 (47.7%) were men and 34 (52.3%) were women, with a mean age of 49.97 ± 4.80 years (range 39–63 years). Among adolescents, 24 (53.3%) were boys and 21 (46.7%) were girls, with a mean age of 15.87 ± 1.77 years (range 12–18 years).

Mean parental body weight was 90.55 ± 18.19 kg and mean height was 169.78 ± 9.69 cm. Mean waist circumference was 106.23 ± 14.08 cm, neck circumference 39.89 ± 4.32 cm, and hip circumference 112.88 ± 11.71 cm. Mean WHR was 0.94 ± 0.09 and mean WHtR was 0.63 ± 0.08. Mean estimated body fat percentage was 38.78 ± 11.91%, while mean BMI was 31.37 ± 5.67 kg/m^2^. According to BMI classification, 9 parents (13.8%) had normal body weight, 16 (24.6%) were overweight, and 40 (61.5%) fulfilled BMI criteria for obesity.

Biochemical and clinical evaluation demonstrated mildly increased mean fasting glucose and blood pressure values, while lipid profile parameters showed substantial interindividual variability. The most commonly reported obesity-related diseases among parents were hyperlipidemia (47.2%), arterial hypertension (30.6%), type 2 diabetes (13.9%), and anxiety/depressive disorders (13.9%).

Mean body weight of the adolescents was 68.53 ± 15.69 kg and mean height was 169.71 ± 10.96 cm. Mean waist circumference was 81.98 ± 11.59 cm, neck circumference 35.84 ± 3.60 cm, and hip circumference 97.87 ± 11.69 cm. Mean WHR was 0.84 ± 0.09 and mean WHtR was 0.48 ± 0.07. Mean BMI was 23.69 ± 4.34 kg/m^2^, while mean BMI percentile was 67.84 ± 29.98. Based on BMI percentile classification, 26 adolescents (57.8%) had a BMI percentile < 85th percentile, 13 (28.9%) had a BMI percentile between the 85th and 95th percentiles, and 6 (13.3%) had a BMI percentile > 95th percentile. Biochemical and clinical parameters were overall within expected ranges, although substantial interindividual variability was observed across several metabolic indices. The key demographic, anthropometric, biochemical, and clinical characteristics of study participants are presented in Table 1.

### 3.2. Associations Between Parental Characteristics and Weight Status in Adolescents

Associations between parental characteristics and adolescent BMI percentile are presented in Table 2. No statistically significant associations were observed between individual parental anthropometric, biochemical, hematological, or clinical parameters and adolescent BMI percentile. Specifically, parental BMI (*p* = 0.901), waist circumference (*p* = 0.713), WHR (*p* = 0.529), WHtR (*p* = 0.555), neck circumference (*p* = 0.840), estimated body fat percentage (*p* = 0.797), fasting glucose (*p* = 0.538), HbA1c (*p* = 0.377), total cholesterol (*p* = 0.601), triglycerides (*p* = 0.564), systolic blood pressure (*p* = 0.936), and diastolic blood pressure (*p* = 0.850) were not significantly associated with adolescent BMI percentile.

**Figure 1:**
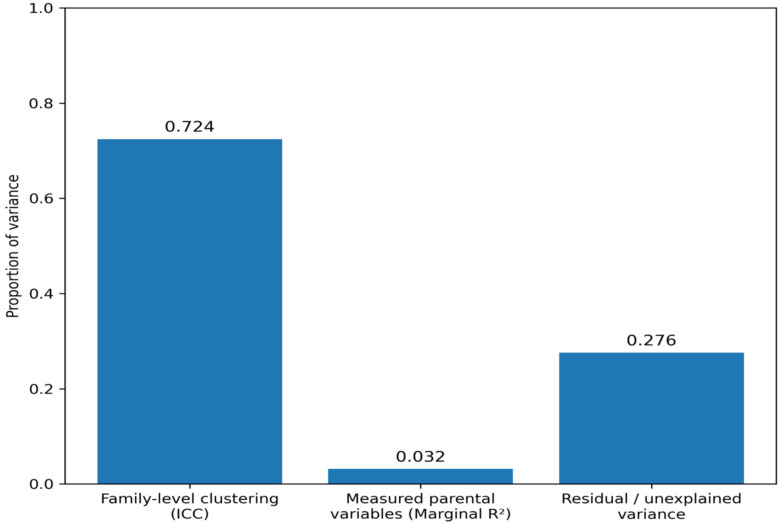
Comparison between family-level clustering and explanatory contribution of measured parental variables in linear mixed models predicting an adolescent BMI percentile. Abbreviations: BMI—body mass index; ICC—intraclass correlation coefficient; R^2^—coefficient of determination.

Despite the absence of statistically significant associations for individual parental variables, strong within-family clustering of adolescent weight status was consistently observed across all models. ICC values ranged from 0.716 to 0.728, indicating that approximately 72–73% of the overall variability in adolescent BMI percentile was attributable to family-level clustering. In contrast, the explanatory contribution of measured parental variables remained limited, with marginal R^2^ values ranging from 0.028 to 0.044, indicating that the fixed parental characteristics explained less than 5% of the observed variability in adolescent BMI percentile. Conditional R^2^ values ranged from 0.720 to 0.736, demonstrating substantially greater explanatory capacity when the family-level clustering effect was incorporated into the models. The family-level random effect remained statistically significant across all analyses (all likelihood ratio test *p* values < 0.001). These findings are graphically illustrated in Figure 1.

## 4. Discussion

The present study investigated the association between parental characteristics and obesity-related parameters in adolescents from families in which at least one parent had obesity. The principal finding was the apparent dissociation between strong family-level clustering of adolescent BMI status and the limited explanatory contribution of routinely measurable parental anthropometric and metabolic characteristics. Here, we expanded beyond conventional BMI-based approaches by evaluating multiple routinely measurable anthropometric indices reflecting central adiposity together with metabolic and biochemical markers within families already enriched for parental obesity. Importantly, by applying hierarchical linear mixed models, we were able to quantify the contribution of individual parental characteristics while simultaneously accounting for family-level clustering. To our knowledge, few studies from Mediterranean populations, and particularly Greece, have combined multidimensional phenotypic assessment with hierarchical analytical approaches in a real-world primary care setting.

The analytical strategy should also be interpreted in light of the exploratory objective of the study. Rather than developing a multivariable prediction model, we sought to evaluate the explanatory contribution of individual routinely measured parental anthropometric and metabolic characteristics relative to the family-level clustering effect. Given the relatively small sample size and the substantial collinearity among several parental anthropometric and metabolic variables, fitting a multivariable mixed-effects model would likely have produced unstable parameter estimates and limited interpretability. The consistency of the findings across all models, with persistently high ICC despite minimal marginal R^2^ values, further supports the robustness of the observed family-level clustering. Larger family-based studies will be required to determine whether combinations of parental characteristics provide incremental explanatory value beyond these shared familial influences.

The magnitude of the observed ICCs also warrants careful interpretation. Previous population-based studies have generally reported modest parent–offspring correlations for BMI, typically ranging from approximately 0.20 to 0.40 [23]. However, these estimates are not directly comparable with the ICCs reported in the present study, which quantify the proportion of total variance attributable to family-level clustering within a linear mixed-effects framework rather than pairwise parent–offspring resemblance. Furthermore, our cohort was intentionally enriched for familial obesity risk by including only families in which at least one biological parent had obesity. Together with the relatively small number of participating families, this selection strategy may have contributed to higher ICC estimates. The 95% CIs for the ICC estimates, derived using parametric bootstrap, ranged from approximately 0.45 to 0.87 across the models. The relatively wide CIs also reflect the uncertainty inherent to the limited number of participating families; however, they consistently support substantial family-level clustering. Accordingly, these findings should be interpreted cautiously and require confirmation in larger, more heterogeneous family-based cohorts.

Although substantial within-family similarity was consistently observed across analyses, no isolated parental variable demonstrated a significant association with adolescent BMI percentile. Shared dietary behaviors, physical activity patterns, sleep characteristics, psychosocial influences, household routines, socioeconomic conditions, and genetic susceptibility may all contribute to the observed clustering patterns [24,25,26]. At the same time, the observation that most adolescents in the present cohort did not fulfill criteria for overweight or obesity suggests substantial heterogeneity in body weight even within apparently high-risk familial environments. This finding may indicate that parental obesity does not translate into a uniform or deterministic phenotype in offspring, but rather interacts with multiple modifying factors that may either amplify or attenuate obesity susceptibility during adolescence. Potential explanations include differences in lifestyle adoption within the same household, varying behavioral responses to parental obesity awareness, age-related variability in adiposity trajectories, and the influence of protective environmental or behavioral factors that were not directly measured in the present study [27]. Additionally, the age of onset of parental obesity was not captured in our study. Consequently, some parents may have developed obesity later in adulthood, reflecting inherited susceptibility patterns that manifest only after adolescence. In such cases, offspring who share a similar predisposition may still exhibit normal weight during adolescence, with the phenotype emerging later in life. The relatively favorable lipid and glycemic profile observed among parents should also be interpreted in the context of the study population. The mean parental age was approximately 50 years, the cohort included both parents with and without obesity, and many participants were receiving routine clinical care for cardiometabolic risk factors. Collectively, these factors may have contributed to a more favorable metabolic profile than might be expected in cohorts composed exclusively of older adults with obesity.

At first glance, the present findings may appear discordant with the extensive literature supporting the familial transmission of obesity. However, they should not be interpreted as contradicting this well-established evidence. Twin and adoption studies have consistently estimated the heritability of BMI to range between approximately 40% and 70%, while large prospective parent–offspring cohort studies have repeatedly demonstrated that parental obesity is among the strongest predictors of childhood and adolescent obesity [28]. More recently, genetically informed analytical approaches, including structural equation modeling, have further shown that a substantial proportion of the covariance between parental and offspring BMI is attributable to inherited genetic factors while also highlighting the contribution of shared environmental influences [29]. Consequently, current pediatric obesity guidelines recognize parental obesity as one of the strongest risk factors for childhood obesity and recommend incorporating family history into obesity risk assessment [30]. Rather than challenging this established evidence, our findings suggest that, within a cohort already enriched for parental obesity, routinely measurable parental anthropometric and metabolic characteristics alone may not adequately capture the complex biological and environmental pathways through which familial obesity susceptibility is transmitted. Genetic susceptibility is likely to operate through polygenic, epigenetic, behavioral, and environmental mechanisms, together with gene–environment interactions, that are not fully reflected by routine parental phenotypic assessment [31,32].

The present findings may have particular relevance for Mediterranean and specifically Greek populations, where high rates of both adult and childhood obesity coexist with ongoing lifestyle transitions and Westernization of dietary patterns, reduced physical activity, and socioeconomic pressures following prolonged economic instability [33]. Within this context, intergenerational shifts in dietary patterns, urbanization, broader social transitions, historical population mobility, and long-standing experiences of social insecurity across previous generations may further influence familial obesity trajectories across generations. Consequently, family-centered prevention and intervention strategies may require broader multidimensional approaches extending beyond simple identification of parental obesity or isolated metabolic risk markers [34].

From a clinical perspective, the present findings reinforce the importance of family-centered approaches to obesity prevention and management. Although our study was not designed to identify specific modifiable determinants of adolescent weight status, the observed family-level clustering supports current recommendations that interventions should target the family environment rather than the adolescent alone. Parents should be encouraged to model healthy dietary habits, promote regular physical activity, reduce sedentary behaviors, ensure adequate sleep, and foster supportive home environments that facilitate sustainable healthy lifestyle behaviors. Such comprehensive family-based strategies remain the cornerstone of obesity prevention and treatment during adolescence [35].

The present study has several strengths, including the family-based design, the inclusion of both parents when available, the use of multiple anthropometric and metabolic parameters beyond BMI alone, and the application of hierarchical statistical models accounting for within-family clustering. In addition, consecutive recruitment from GP registries enhances the real-world relevance of the findings. However, important limitations should be acknowledged. Information on the age of onset of parental obesity was unavailable, potentially limiting the identification of inherited obesity susceptibility that may become clinically apparent only later in life. The cross-sectional design precludes causal inferences. The relatively small number of participating families limits the precision of the estimated model parameters and reduces the ability to detect modest associations between individual parental characteristics and adolescent weight status. Consequently, the absence of statistically significant associations should not be interpreted as definitive evidence that there is no relationship. Nevertheless, the study consistently demonstrated a robust family-level clustering effect, suggesting that the principal findings are unlikely to be solely attributable to insufficient statistical power. Larger prospective family-based studies are warranted to validate these observations and to determine whether additional parental anthropometric, metabolic, behavioral, or genetic characteristics account for the observed familial clustering. An additional limitation relates to the recruitment strategy. By restricting eligibility to families in which at least one biological parent had obesity, the variability of parental anthropometric characteristics was intentionally reduced. Although this approach enriched the cohort for familial obesity risk and aligned with the study objective of investigating within-family variability in adolescent weight status, it may also have attenuated associations between individual parental characteristics and adolescent weight status because of the restricted range of parental anthropometric measures. Consequently, our findings should not be interpreted as evidence that parental adiposity is unimportant. Rather, within this already high-risk population, the routinely measured parental anthropometric and metabolic characteristics evaluated did not demonstrate statistically significant associations with adolescent weight status and therefore could not account for the observed within-family variability. Whether this reflects a true limited explanatory contribution or the effects of restricted variability and limited statistical power requires confirmation in larger studies.

Body fat percentage was estimated using the US Navy circumference formula rather than reference methods such as dual-energy X-ray absorptiometry or validated bioelectrical impedance analysis. Although this approach is practical and widely applicable in clinical and primary care settings, it is less accurate in adults with obesity and may have introduced measurement error, potentially attenuating associations involving body fat percentage. Furthermore, the use of glucose-lowering, lipid-lowering, and antihypertensive medications was not accounted for in the analyses and may have favorably influenced the measured metabolic parameters. Consequently, pharmacological treatment represents a potential confounder that should be considered when interpreting the relatively favorable metabolic profile of the parent cohort. In addition, the absence of detailed information regarding dietary habits, physical activity, sedentary behavior, sleep characteristics, psychosocial variables, and socioeconomic status limits mechanistic interpretation of the observed clustering patterns. Importantly, the aim of the present study was not to investigate the already well-established multifactorial etiology of obesity, but rather to examine whether routinely measurable parental anthropometric and metabolic characteristics could explain within-family similarity in adolescent weight status in this specific real-world population. In this context, the substantial residual unexplained variance suggests that the routinely measured parental anthropometric and metabolic characteristics evaluated in the present study were insufficient to fully explain the observed family-level clustering. However, the present study was not designed to determine whether this reflects the limited explanatory value of these routinely measured characteristics or the influence of restricted variability and limited statistical power. The underlying mechanisms remain uncertain and may include genetic inheritance, shared household environment, behavioral factors, gene–environment interactions, epigenetic influences, or other unmeasured determinants. These possibilities cannot be distinguished by the present study design.

## 5. Conclusions

In conclusion, the findings of the present study suggest that strong family-level clustering of adolescent weight status may persist despite the absence of statistically significant associations with the individual parental anthropometric and metabolic characteristics evaluated. These observations should not be interpreted as evidence that parental anthropometric characteristics are unrelated to adolescent weight status, nor do they establish the mechanisms underlying the observed familial clustering. Rather, within this selected high-risk cohort, the routinely measurable parental characteristics examined explained only a limited proportion of the observed within-family variability. We acknowledge that the relatively small sample size and the restricted variability of parental anthropometric characteristics may also have contributed to the uniformly non-significant associations. Larger prospective longitudinal family-based studies in more heterogeneous populations, integrating behavioral, environmental, genetic, epigenetic, and metabolic data, are warranted to validate these findings and elucidate the determinants of familial obesity susceptibility.

## Figures and Tables

**Table 1:** Demographic, anthropometric, biochemical, and clinical characteristics of participants.

Variable	Parents (*n* = 65) Mean ± SD or *n* (%)	Adolescents (*n* = 45) Mean ± SD or *n* (%)
Age (years)	49.97 ± 4.80	15.87 ± 1.77
Female sex	34 (52.3)	21 (46.7)
Weight (kg)	90.55 ± 18.19	68.53 ± 15.69
Height (cm)	169.78 ± 9.69	169.71 ± 10.96
BMI (kg/m^2^)	31.37 ± 5.67	23.69 ± 4.34
BMI percentile	—	67.84 ± 29.98
Waist circumference (cm)	106.23 ± 14.08	81.98 ± 11.59
Neck circumference (cm)	39.89 ± 4.32	35.84 ± 3.60
Hip circumference (cm)	112.88 ± 11.71	97.87 ± 11.69
WHR	0.94 ± 0.09	0.84 ± 0.09
WHtR	0.63 ± 0.08	0.48 ± 0.07
Estimated body fat percentage (%)	38.78 ± 11.91	—
Normal body weight	9 (13.8)	26 (57.8)
Overweight	16 (24.6)	13 (28.9)
Obesity	40 (61.5)	6 (13.3)
Hematocrit (%)	42.09 ± 4.18	41.23 ± 2.73
Platelets (×10^3^/μL)	252.22 ± 60.84	262.89 ± 44.51
SGOT (U/L)	24.88 ± 15.13	22.36 ± 6.56
SGPT (U/L)	29.46 ± 22.62	20.73 ± 10.19
Creatinine (mg/dL)	0.83 ± 0.15	0.76 ± 0.14
eGFR (mL/min/1.73 m^2^)	97.89 ± 11.41	128.33 ± 13.53
Total cholesterol (mg/dL)	199.83 ± 43.91	148.56 ± 27.67
HDL-C (mg/dL)	50.37 ± 12.15	50.91 ± 12.83
LDL-C (mg/dL)	124.97 ± 38.05	81.60 ± 24.99
Triglycerides (mg/dL)	130.06 ± 74.66	76.00 ± 35.51
Fasting glucose (mg/dL)	101.71 ± 14.76	91.36 ± 7.76
HbA1c (%)	5.67 ± 0.81	5.15 ± 0.30
FIB-4	0.97 ± 0.34	—
Systolic blood pressure (mmHg)	133.26 ± 16.82	117.44 ± 12.73
Diastolic blood pressure (mmHg)	85.74 ± 12.86	68.67 ± 12.52

Abbreviations: BMI—body mass index; eGFR—estimated glomerular filtration rate; HbA1c—glycated hemoglobin; HDL-C—high-density lipoprotein cholesterol; LDL-C—low-density lipoprotein cholesterol; SD—standard deviation; SGOT—serum glutamic oxaloacetic transaminase; SGPT—serum glutamic pyruvic transaminase; WHR—waist-to-hip ratio; WHtR—waist-to-height ratio. “—” Not estimated in this group.

**Table 2:** Associations between parental characteristics and adolescent BMI percentile using linear mixed models.

Parental Variable	B	SE	*p* Value	ICC	Lower 95% CI	Upper 95% CI	Marginal R^2^
BMI	0.11	0.89	0.901	0.727	0.484	0.864	0.029
Weight	0.05	0.32	0.873	0.727	0.480	0.865	0.030
Height	−0.46	0.49	0.340	0.716	0.474	0.858	0.044
Waist circumference	0.11	0.30	0.713	0.725	0.480	0.863	0.029
Neck circumference	−0.13	0.65	0.840	0.727	0.483	0.866	0.028
Hip circumference	0.01	0.28	0.457	0.727	0.489	0.866	0.029
WHR	23.82	37.47	0.529	0.722	0.474	0.860	0.032
WHtR	−18.06	30.40	0.555	0.721	0.480	0.860	0.031
Estimated body fat percentage	−0.12	0.46	0.797	0.726	0.481	0.864	0.029
Fasting glucose	−0.12	0.20	0.538	0.721	0.478	0.864	0.029
HbA1c	3.57	4.02	0.377	0.722	0.476	0.862	0.033
Total cholesterol	−0.07	0.13	0.601	0.724	0.480	0.864	0.033
HDL-C	−0.19	0.51	0.588	0.725	0.487	0.864	0.034
LDL-C	−0.05	0.17	0.782	0.727	0.489	0.864	0.030
Triglycerides	0.02	0.08	0.564	0.728	0.486	0.864	0.029
Hematocrit	0.34	0.69	0.628	0.723	0.478	0.862	0.030
Platelets	−0.00	0.04	0.903	0.728	0.481	0.865	0.029
SGOT	0.19	0.20	0.333	0.720	0.465	0.861	0.038
SGPT	0.18	0.21	0.407	0.720	0.472	0.863	0.034
Creatinine	7.07	10.18	0.490	0.721	0.479	0.862	0.035
eGFR	−0.19	0.36	0.600	0.724	0.486	0.863	0.033
FIB-4	−2.41	3.28	0.633	0.725	0.488	0.864	0.032
Systolic blood pressure	−0.02	0.08	0.936	0.728	0.489	0.865	0.029
Diastolic blood pressure	−0.06	0.31	0.850	0.728	0.483	0.866	0.029

Abbreviations: B—unstandardized regression coefficient; BMI—body mass index; CI—confidence interval; eGFR—estimated glomerular filtration rate; FIB-4—fibrosis-4 score; HbA1c—glycated hemoglobin; HDL-C—high-density lipoprotein cholesterol; ICC—intraclass correlation coefficients; LDL-C—low-density lipoprotein cholesterol; SE—standard error; SGOT—serum glutamic oxaloacetic transaminase; SGPT—serum glutamic pyruvic transaminase; WHR—waist-to-hip ratio; WHtR—waist-to-height ratio.

## Data Availability

The data presented in the study are available on request from the corresponding author. The data are not publicly available due to privacy restrictions of the Greek National Health System.
